# Impact of Ag_2_O on physical and electrical properties of Li_2_O-MgO-Bi_2_O_3_-SiO_2_ glass system as promising solid state electrolytes

**DOI:** 10.1038/s41598-025-87463-0

**Published:** 2025-02-07

**Authors:** Hany A. Abo-Mosallam, Samia E. Ibrahim

**Affiliations:** 1 Glass Research Department, National Research Centre, El Buhouth St, Dokki, Cairo, 12622 Egypt; 2 Physics Department, Faculty of Science, Menoufia University, Shebin El-Koom, Menoufia, 32511 Egypt

**Keywords:** Glass, Ag_2_O, Bismuth-silicate, Structure, Electrical, Solid electrolytes, Applied physics, Condensed-matter physics

## Abstract

The intrinsic aim of this work is to understand the influence of composition change on the physical-electrical parameters of glasses with the composition (20-Y) Li_2_O-YAg_2_O–20MgO–10Bi_2_O_3_-50SiO_2_ (Y = 0.25, 0.5, 1.0, 2.0 and 4.0 mol%). The results of the physical parameters indicate a growth in the strength of the glass structure with the increase replacement of Li_2_O by Ag_2_O. However, the decrease in loss factor ε″*,* and AC conductivity *σ*_*ac*_ of glasses may be attributed to the decrease in the space charge polarization (scp). AC conductivities exhibit simple power law variation. The s values essentially lie in the range of 0.67–0.92, which is typical of conductors in Jonscher regime. Dielectric properties (constant ε′, loss ε″ and ac conductivity σ_ac_, over a range of frequency 100–100 kHz and temperature RT (Room Temperature)–200 °C and frequency exponent s) of these glasses have been studied The results indicate a great potential of the prepared glasses as promising materials that can be used for solid state electrolytes.

## Introduction

Non-crystalline substances are one of the greatest unique and important materials. Glass materials have an extensive range of physical and chemical properties that qualify them for use in new and advanced applications^1–3^. Glass containing bismuth oxide is one of the most distinguished materials due to its many and promising applications^4,5^. Especially, bismuth silicate glass which is used in many applications because it has great physical and optical properties^4^. As a result of the high polarizability of the Bi^3+^ ion, it effectively affects the structural integrity of the amorphous lattice. As a result of the presence of silica as a building block of the glass network, bismuth may be present in two different valences modifier units [BiO_6_] and network pyramidal former units [BiO_3_], which effectively affect the electrical properties of the resulting glass^5–8^.

Recently, ionic conductors have become of great interest and important industrial applications, such as in solid state batteries^9,10^. Glass materials are considered one of the most important and leading materials for use in the field of solid electrolytes in the manufacture of electrical battery^11^. Currently, bismuth silicate glass is used in many advanced applications^12^. Despite its many advantages, especially the physical and optical ones, the low electrical conductivity of bismuth silicate glass is considered the most prominent of its disadvantage^13^. Several studies were conducted on bismuth silicate glass by adding alkali metals and alkaline earth metals, which significantly improved the electrical properties of this glassy system^1,14–16^. When bismuthate glasses contain Li^+^ions, this leads to improvement of their electrical properties^6,17,18^. The solubility of silver oxide in varying amounts in the glass melt is very important and works to create new materials with diverse physical, electrical and biological properties^19,20^. The ionic conductivity of glasses can be improved by adding silver compounds to the glass composition^21–23^. Improving the ionic conductivity could be achieved by modification of amorphous material network using glass modifier oxides like Ag_2_O. Glass matrices depolymerize due to some factors such as the introduction of a group of modified oxides on the glass network leads to the breaking of oxygen bonds and the presence of large amounts of non-bridging oxygen ions and significantly improves the ionic conductivity of the glass systems^23,24^. Alkali-Bi_2_O_3_-SiO_2_non-crystalline materials characterized by high ionic-conductivity which made it enter into various modern technological applications in varied fields such as solid state electrolytes^25^, high energy batteries and sensors^1,15^. Recently, many researchers and scientists in the field of materials science have been interested in glass systems based on silver borate. This is due to the high ionic conductivity of these glass systems due to the presence of free silver ions and consequently their wide applications in the field of solid electrolytes^26,27^. The effect of silver in different glass systems used for solid electrolytes application was studied^23,28,29^. The glasses doped with silver can be considered potential candidates for solid electrolytes. So, the main goal of this study is to define the impact of substituting of Li_2_O by Ag_2_O on the structural and physic-electrical behavior of the prepared glass materials having compositions (20-Y) Li_2_O-Y Ag_2_O-20 MgO-10Bi_2_O_3_−50 SiO_2_ (where Y = 0.25, 0.5, 1.0, 2.0 and 4.0 mol %) to investigate the relationship of the change in the glass compositions and the resulting properties.

## Experimental technique

### Glass synthesis

Glass specimens with a composition consisting of (20-Y) Li_2_O—Y Ag_2_O −20 MgO-10Bi_2_O_3_−50 SiO_2_ (where Y = 0.25, 0.5, 1.0, 2.0 and 4.0 mol %) were designed and synthesized by melt-quenching techniques. The glass batches were synthesized from high purity chemicals of lithium carbonate (Li_2_CO_3_), magnesium oxide (MgO), bismuth oxide (Bi_2_O_3_), silver nitrate (AgNO_3_) and quartz (SiO_2_) as shown in Table 1. The designed batches were mingled in the rotator machine for 90 min to ensure the homogeneity of the mixtures. Then the batches were putted in an alumina crucible and melted in the melting oven at a temperature ranging from 1100 to 1250 °C. After completing the melting process and making sure that there are no gas bubbles in the melt. The molten glass then poured into pre-heated stainless steel molds of different shapes. Then the cast glass samples were placed in an annealing furnace for approximately 2 h at 300–350 °C to relieve the residual stresses and strains.

**Table 1:** The composition of the prepared glasses.

Sample ID	Oxide Mol %					Melting Temperature ( °C)
	**Li**_**2**_**O**	**Ag**_**2**_**O**	**MgO**	**Bi**_**2**_**O3**	**SiO**_**2**_	
GAg1	20	0	20	10	50	1100
GAg 2	19.75	0.25	20	10	50	1125
GAg 3	19.5	0.5	20	10	50	1150
GAg 4	19	1	20	10	50	1175
GAg 5	18	2	20	10	50	1200
GAg 6	16	4	20	10	50	1250

### Glass characterization

Using a Philips PW 1730 X-ray diffractometer, XRD analyses were carried out on the produced specimens to ascertain their amorphous nature. The Fourier-transform infrared spectroscopy (FTIR) of the synthesized Ag_2_O-doped lithium magnesium bismuth silicate glasses were measured at ambient temperature in the wavenumber range 400–2000 cm^−1^ by a FTIR spectrometer by (Jasco-6100, Japan) by the potassium bromide disc technique at a ratio of 1:100. The prepared glasses were examined as pulverized powders which were mixed with KBr with the ratio 1:100mg glass powder to KBr, respectively. The IR spectral measurements were carried out directly after making the discs.

The density, ρ, of prepared specimens was calculated based on standard Archimedes’ method. To determine the density, six pieces of glass were used for each sample, after ensuring that they were free of air bubbles. The relative masses of non-crystalline pieces in air and distilled water as the immersion liquid were weighted using an analytical balance (Radwag AS 220.R2 PLUS Analytical Balance, Polanda) with a correctness of ± 0.0001 mg. Then the density was calculated using Eq. 1. Where W_A_ and W_L_ are the glass specimens masses in air and immersion liquid respectively. The ρ_w_ is the density of distilled water at ambient temperature (1.0 g/cm^3^)^30^ . The molar volume V_m_ of the prepared specimens was determined from the densities of the samples as shown in Eq. 2. where x_i_ is the molar fraction, M_i_is the molecular weight of each component and ρ is the specimen density^31^ . The oxygen packing density (OPD) was calculated using Eq. 3^32^ . Where N_O_ is quantity of oxygen’s in the glass system. The oxygen molar volume (V_o_) was calculated by utilizing the Eq. 4^33^ . The field strength (F) was measured by utilizing the Eq. 5^33^. While *R*_*p*_ and *z* are the ionic radius and atomic number of silver ion respectively. The ionic radius *R*_*p*_ was calculated by utilizing the Eq. 6^34^.

1 $$\rho{\text{ = W}_{\text{A}}/(W}_{\text{A}} - W_{{\text{L}}} {\text{)}}\times\rho _{w}$$

2 $$V_{{\text{m}}} = \sum {\text{X}}_{{\text{i}}} {\text{M}}_{{\text{i}}} {/}\rho$$

3 $$\text{OPD}=1000 N_{{\text{o}}}/V_{{\text{m}}}$$

4 $$V_{{\text{o}}}=V_{{\text{m}}}/\sum {\text{X}}_{{\text{i}}} {\text{M}}_{{\text{i}}} $$

5 $${\text{F = Z/r}}_{{\text{p}}}^{{2}}$$

6 $${\text{R}}_{{\text{p}}} { = 1/2(}\pi {\text{/6N)}}^{{1/3}}$$

### Electric and dielectric measurements

For electrical measurements, the prepared samples were polished to obtain disk shape samples with 2 mm thickness and 5 mm diameter. Good electrical contact was achieved by depositing the faces of the samples with silver paste. The dielectric parameters like the impedance (Z), capacitance (C) and dielectric loss factor (tan δ) were measured using (Hioki 3520 LCR HiTester Meter Bridge). In the frequency ranges (40 Hz—10^5^ Hz), the measurements were recorded at different temperatures in the range of (RT—200 °C). This LCR bridge measures the frequency (f), capacitance (C), impedance (Z), phase angle (Φ), and dissipation factor (tan δ). The dielectric constant (ε′) and dielectric loss (ε′′) can be calculated.

## Results and discussion

### Glass formation and characterization

#### Glass formation

**Fig. 1:**
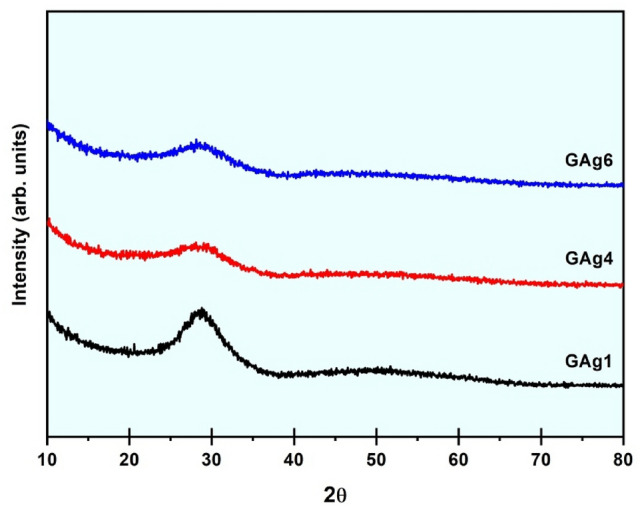
The XRD pattern of the (GAg1), (GAg4) and (GAg6) specimens.

As seen in Fig. 1, X-ray diffraction was used to confirm the glassy character of the prepared specimens, the samples GAg1 (free of Ag_2_O) , GAg4 (with Ag_2_O 1 mol%) and GAg6 ( with high Ag_2_O content 4 mol %) were investigated. The XRD result obviously supports the glassy structure of all the prepared specimens of (20-Y) Li_2_O—Y Ag_2_O −20 MgO-10Bi_2_O_3_−50 SiO_2_ (where Y = 0.25, 0.5, 1.0, 2.0 and 4.0 mol %) as shown in Fig. 1. The XRD data showed a broad hump about 20° ≤ 2θ ≤ 30° without any sharp peaks, confirming the amorphous nature of the samples.

#### FTIR spectroscopy

**Fig. 2:**
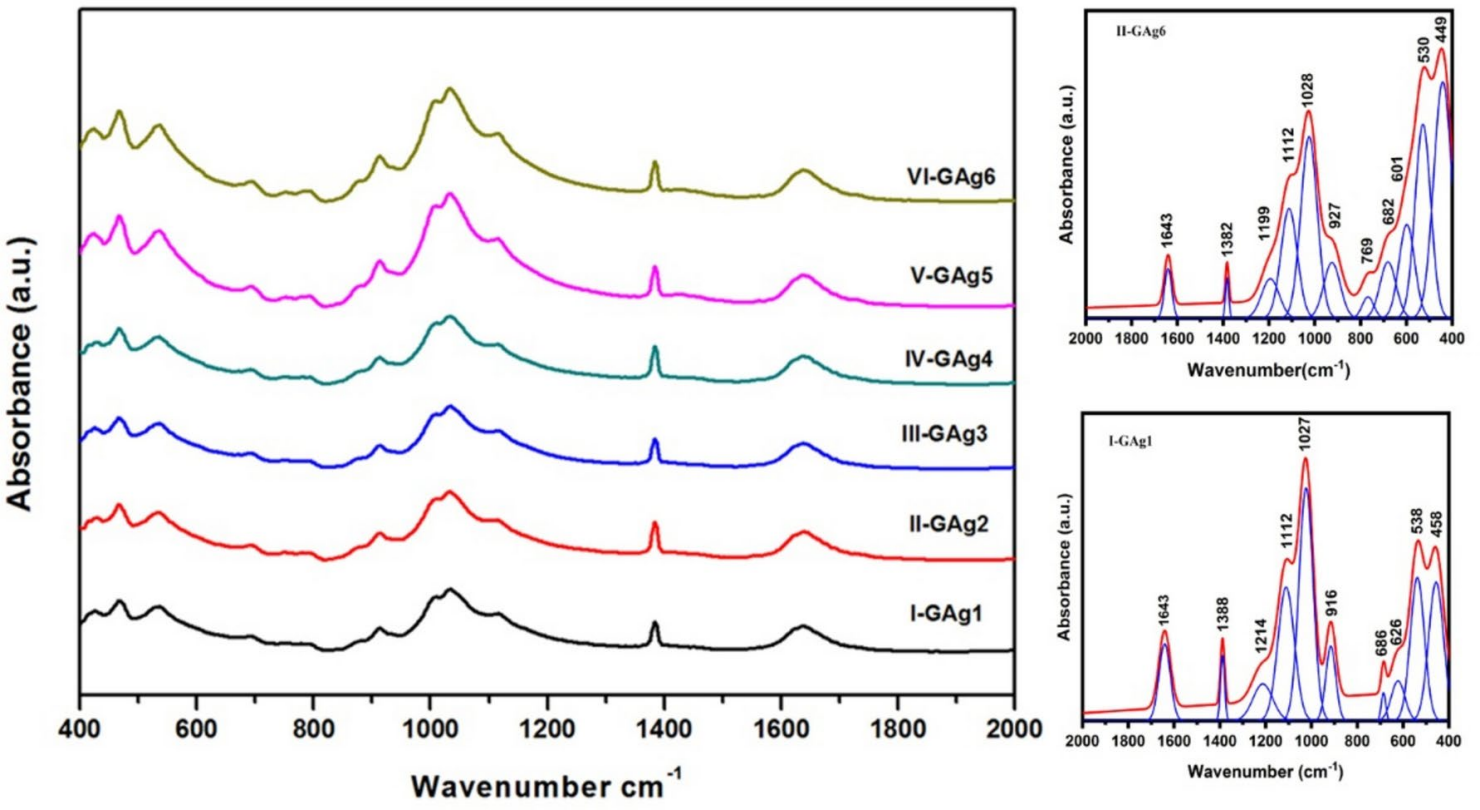
The FTIR absorbance spectra for the synthesized specimens recorded in the spectral range 400 and 2000 cm^−1^, the deconvoluted spectra for the GAg1, and GAg6 specimens (right).

The Fourier transform infrared (FTIR) technique^1,35^ used to examine the alteration of the vibrational types of the diverse groups in the glass specimens. From here, the fundamental changes that occurred in the internal structure of the prepared glass as a result of the replacement of Li_2_O with Ag_2_O were identified by the FTIR technique. Fig. 2 presents the obtained FTIR bands of the examined glasses based on Li_2_O-MgO-Bi_2_O_3_-SiO_2_ system modified by the Ag_2_O/Li_2_O replacement. The absorption spectra of the glass specimens listed at 25 °C in the range 2000 –400 cm^−1^ are displayed in Fig. 2. From the obtained IR results, a slight difference in the positions of the absorption bands in the mid- and near-infrared spectra of the prepared glass was observed compared to the absorption bands of glasses based on silica in the composition. This phenomenon can be attributed to the presence of the bismuth oxide as heavy metal and modifying cations such as lithium and magnesium^1,36^. The FTIR curves show that there are three main absorption regions in the 1300–1800 cm^−1^, 800–1300 cm^−1^, and 400–700 cm^−1^ as shown in Fig. 2.

The FTIR spectra show some wide bands, which are formed as a result of the overlapping of separate close peaks. Therefore, the infrared spectrum is deconvoluted in order to know the exact peak values of the synthesized glass specimens using curve fit Fig. 2. The FTIR spectra of base glass GAg_1_ exhibited the subsequent spectral features: At the 700–400 cm^−1^ wavenumber regions, four bands are present at about 458 cm^−1^, 538 cm^−1^, 626 cm^−1^ and 686 cm^−1^, respectively. At the 800–1350 cm^−1^ wavenumber area there are four bands at about 916 cm^−1^, 1027 cm^−1^, 1112 cm^−1^ and 1214 cm^−1^ respectively. Finally, at the 1300–1800 cm^−1^ wavenumber region there are only two peaks at about 1388 and 1643 cm^−1^. The peaks in the region 400–700 cm ^− 1^ are attributed to Bi-O in BiO₆ octahedral/BiO₃ pyramidal^37^], O–Si–O bending, and Si–O-Si bending types of vibration^38,39^. However the peaks in the range of 800–1300 cm^−1^ are attributed to the stretching vibration of the SiO_4_tetrahedral with the dissimilar quantity of (BO) atoms^40^. Lastly, at the 1300–1800 cm^−1^ wavenumber region there are two peaks at 1388 cm^−1^ and 1643 cm^−1^, respectively, which can be attributed to the vibrations resulting from carbonate groups^4,41^and vibrations of water molecule^4,42^ respectively. From the results of the FT-IR spectra, it is clear that by Ag_2_O/Li_2_O replacements up to 1.0 mol % there is no difference in the patterns. When the quantity of Ag_2_O is augmented from 1.0 to 4 mol %, the strength of the prepared glasses increases. This result is fully consistent with previous research where adding Ag_2_O in the glasses resulted in a remarkable decrease in the non-bridging oxygen’s (NBO) and increases the strength of the glass network^43–46^.

#### Physical parameters

**Fig. 3:**
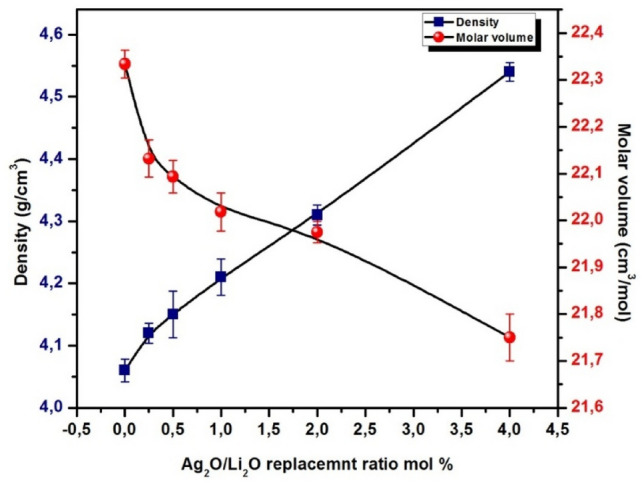
The density and molar volume for the synthesized sample as a function of Ag_2_O/Li_2_O replacement ratio.

Density ρ is one of the vital and significant physical parameters for materials. For glass material, density is important and necessary, as it greatly helps to understand the fundamental variations in the internal construction of the glass as a result of the alteration in compactness and strength^3,47^. The ρ of the amorphous material is affected by many factors, the most important are the glass composition, annealing treatment regime, and internal compactness of structure^3^. Therefore, in this study, the ρ and V_m_ of the synthesized materials were resoluted to understand the effect of the modification in composition on the glass structure formed as shown in Fig. 3, and Table 2.

**Table 2:** The physical parameters of the investigated glasses.

**Sample** **ID**	**M** **(g/mol)**	**ρ (g/cm**^**3**^**)** ** ± 0.001**	**V**_**m**_** (cm**^**3**^**/mol)** **( (± 0.01)**	**OPD** **(gm atml**^**−1**^**)**	**V**_**O**_ **(cm**^**3**^**/mol)**	**R**_**p**_ **(10**^**–8**^** ions/cm**^**3**^**)**	**F** **(10**^**15**^** cm**^**2**^**)**
GAg1	90.68	4.06	22.78	76.11	13.40	–––	––––
GAg 2	91.18	4.12	22.13	76.81	13.02	9.87	4.82
GAg 3	91.68	4.15	22.09	76.95	12.99	7.83	7.66
GAg 4	92.70	4.21	22.02	77.21	12.95	6.21	11.42
GAg 5	94.71	4.31	21.98	77.36	12.92	4.92	19.43
GAg 6	98.75	4.54	21.75	78.16	12.79	3.90	30.92

The density results of the prepared glass specimens showed that it increased with replacement of Li_2_O by Ag_2_O. These results may be attributed to the clear difference between the molecular weight and density of Ag_2_O and Li_2_O. Silver oxide has (ρ = 7.14 gcm^−3^ and Molar mass = 231.74 g/mol) while lithium oxide has ρ = 2.01 g/cm^3^and Molar mass = 29.88 g/mol^48^. The influences of substituting lithium with silver on changes in *V*_m_ of the prepared glasses are potted in Fig. 3 and Table 2. According to the *V*_m_ calculation, when Ag_2_O is added into the glass composition instead of Li_2_O up to 4.0 mol%, the molar volume decreased from 22.78 to 21.75 cm^3^/mol. It is scientifically proven that a significant change in the molar volume of non-crystalline material is closely compatible with the change in its internal network structure^47,49^. The decrease in the V_m_of the prepared material is due to the large molar volume of silver oxide, which fills the spaces in the glass network^50^. Also increase in the compactness of the structure and formation the bridging oxygen instead of non-bridging oxygens (NBO)^43^.

**Fig. 4:**
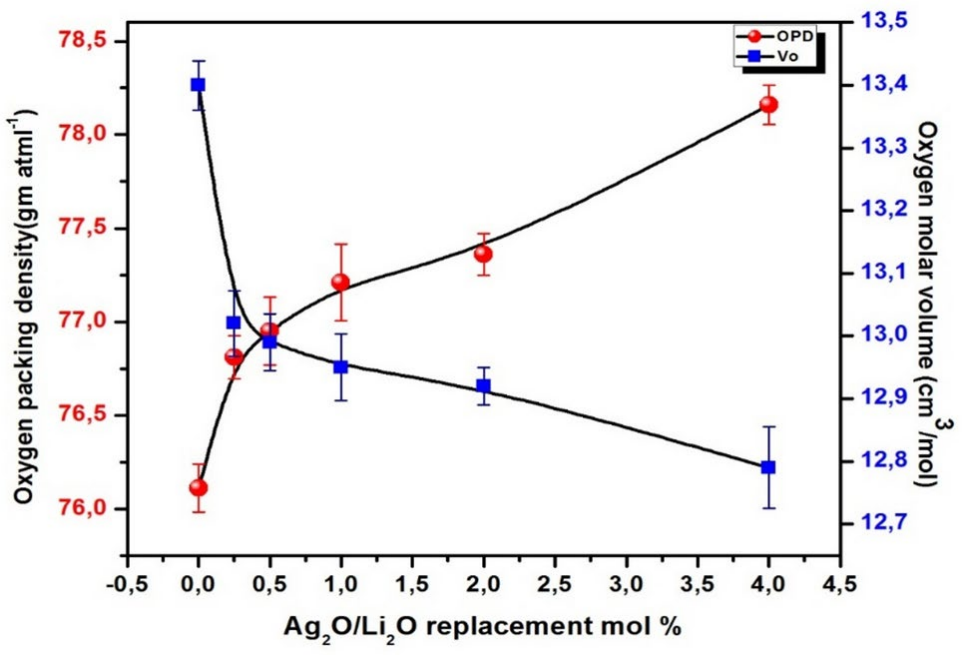
The oxygen packing density and The oxygen molar volume for the prepared glass samples as a function of Ag_2_O/Li_2_O replacement ratio.

The impact of substituting Li_2_O with Ag_2_O on both oxygen packing density (OPD) and oxygen molar volume (V_o_) was studied as shown in Table 2 and Fig. 4. It is clear from the results that the Ag_2_O/Li_2_O replacement process has led to an increase in the OPD from 76.11 to 78.16 (gm atml^**−1**^). On the contrary, the V_o_ parameter decreased from 13.40 to 12.79 (cm^3^/mol) as shown in Fig. 4 and Table 2). The oxygen packing density OPD shows an increase with the increase of Ag_2_O content. In parallel, the replacement of Li_2_O by Ag_2_O lowers the oxygen molar volume. In the studied glasses, oxygen packing density (OPD) increases with Ag_2_O doping from 76.11 to 78.16 which signifies the shrink of the oxygen network in the glass matrix, implying to strengthening of the glass structure^51^. Oxygen molar volume (OMV) is determined by using Eq. 4 which illustrates the OMV decreases from 13.40 to 12.79 cm^3^/mol, this infers that it introduces less free volume in glass structure^51^.

**Fig. 5:**
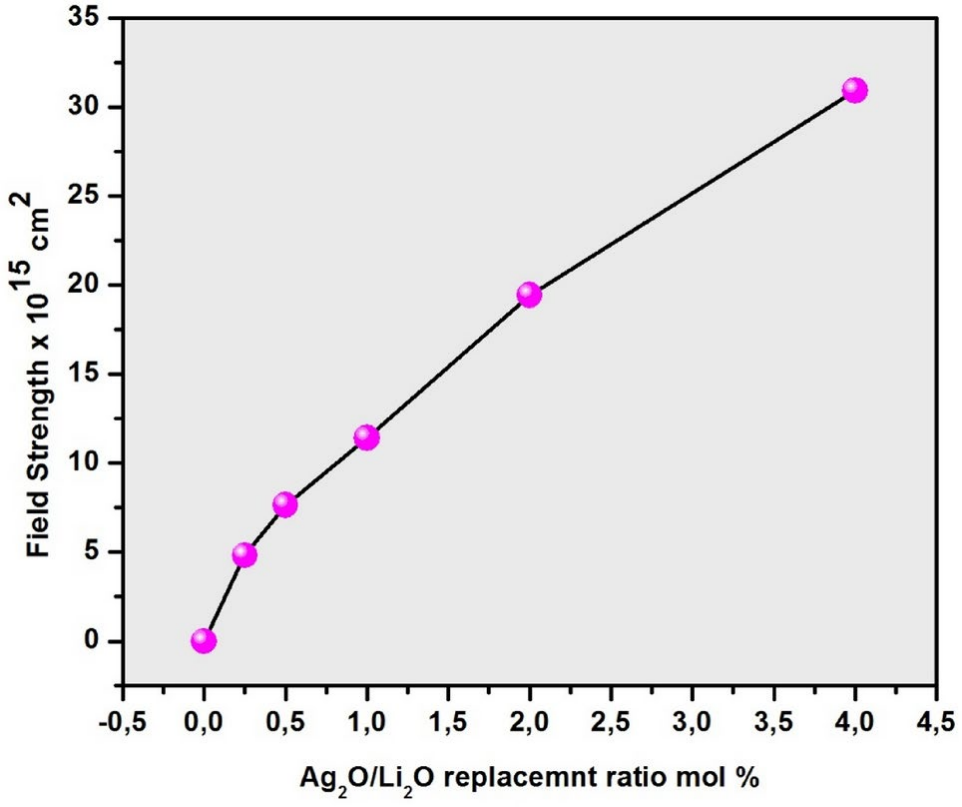
The calculated field strength (F) of the prepared glass samples as a function of Ag_2_O/Li_2_O replacement ratio.

The (OPD) parameter designates the distribution of oxygen atoms in the prepared glass internal structure. These results can be attributed to the increase in the strength of the synthesized amorphous network achieved by adding and increasing the concentration of silver oxide. The increased strength and toughness of the glass network is reflected in the OPD parameter and leads to an increase in its values^52–54^ . On the other side, the modulus field strength data of the prepared glasses are listed in Table 2 and are depicted graphically in Fig. 5 as a function of Ag_2_O content.

The results show that (F) rises with growing amounts of Ag_2_O as shown in Fig. 5. The increase in field strength may be attributed to compactness of glass structure increases.

### Electrical properties

#### Frequency dependent electrical properties

The Numerous researchers have examined the AC conductivity in various non-crystalline substances, proposing different models to explain its relationship with temperature and frequency. To interpret impurity conduction in n-type silicon, Polak and Geballe^55^introduced a quantum mechanical tunneling model, where the exponent s is independent of temperature but varies with frequency. In their study of AC conductivity across a wide range of materials, Hill and Jonscher^56^found that the exponent s typically ranged from 0.5 to 1 at room temperature. Elliot^57^ developed the correlated barrier hopping (CBH) model for glassy semiconductors, suggesting that bipolaron hopping (the movement of two electrons between charged defects D^+^ and D) could explain the frequency-dependent conductivity in glass.

The conductivity (σ_T_) depends on frequency based on the universal dynamic response as deducted from Eq. (7):

7 $${\sigma }_{ac}={\sigma }_{T}-{\sigma }_{dc}=A{\omega }^{s}$$

**Fig. 6:**
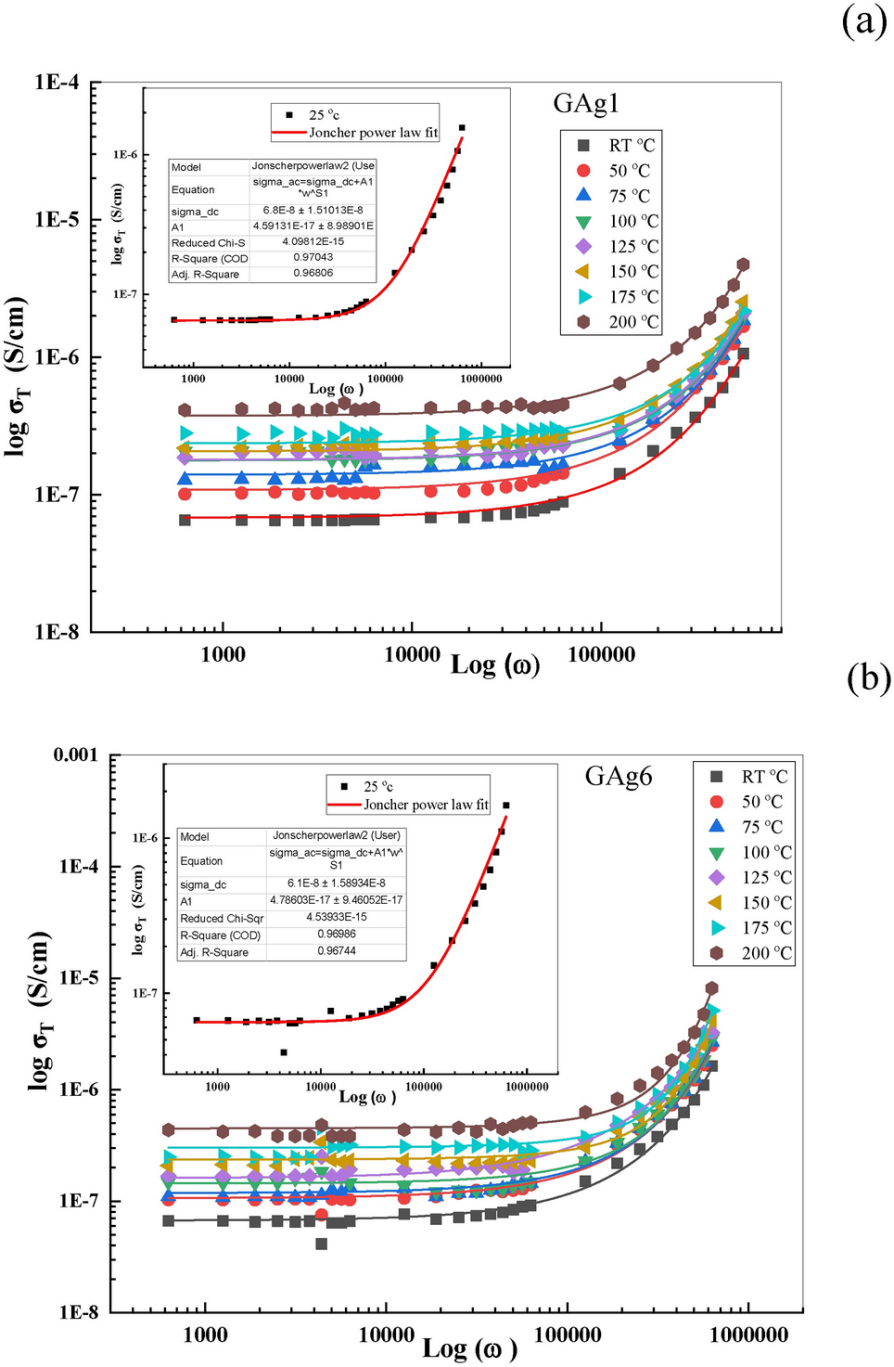
log σ_T._ as a function of log (ω) at different T for (a) GAg1 and (b) GAg6.

σ_dc_ = the independent frequency dc conductivity, s = power law exponent/frequency exponent (related to the degree of correlation among moving ions), and A = constant (temperature-dependent parameter). The plot of log (*σ*_T_) vs. log (*ω*) in the range of 10^2^ Hz to 10^5^ Hz at (RT–200 °C) is shown in Fig. 6 (a, b) for GAg1 and GAg6 samples respectively as an example. As evident from that figure, the *σ*_T_ behavior adopted by our samples shows an increase of conductivity with increasing frequency (f) and this stems from the hopping mechanism that occurs on applying the electrical field.

**Fig. 7:**
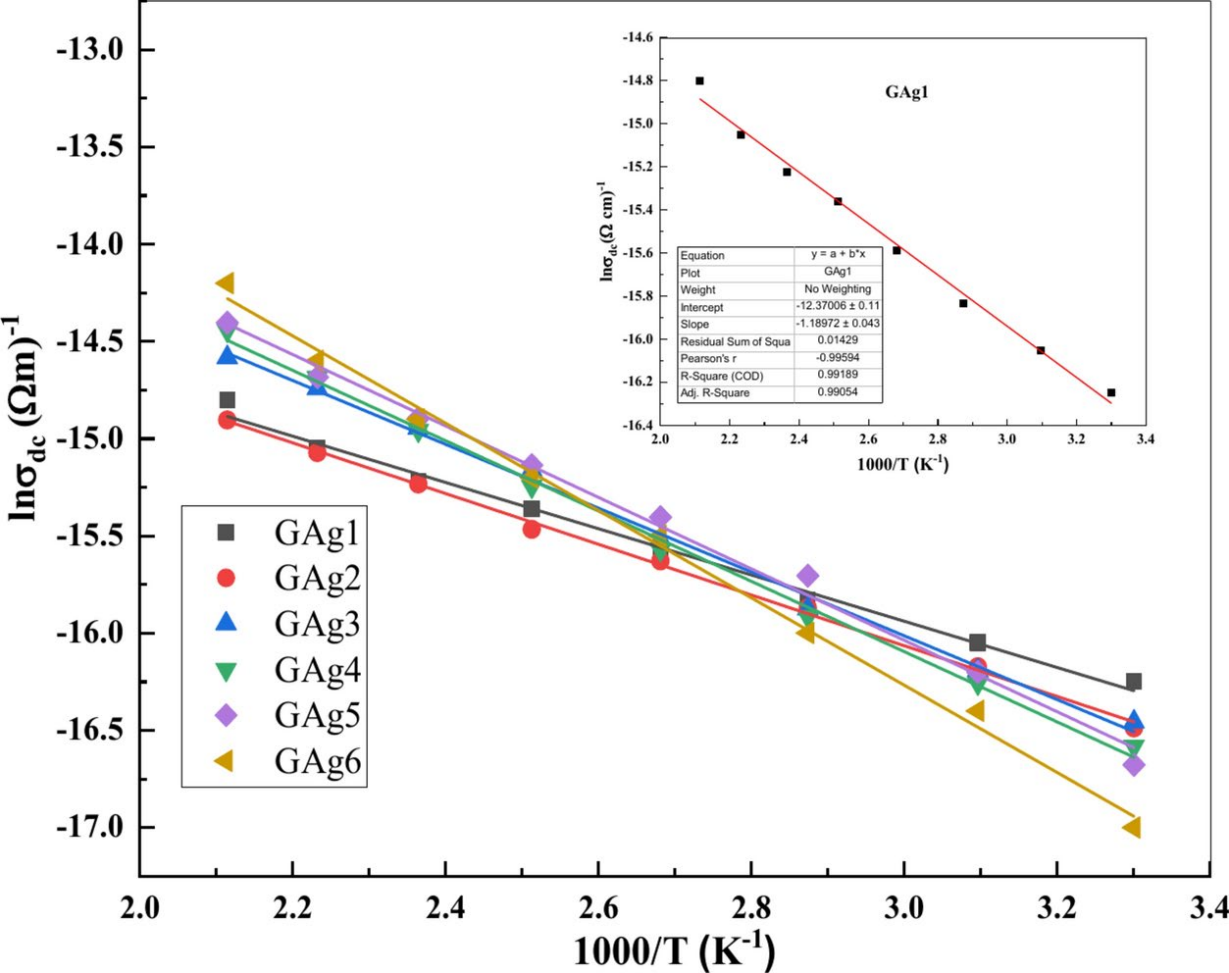
Variation of ln σ_dc_ vs. 1000/T for all glass samples.

We can obtain the “σ_dc_” values by fitting Fig. 6 using Jonscher,s power law and the obtained value has been plotted as a function of 1000/T for all the glass samples (Fig. 7).

The plot of (log σ_dc_) with reciprocal temperature for all glass samples outlined in the range RT–200°C displays straight line nature (Fig. 7). The obtained values of σ_dc_ are thermally activated with increasing temperature and demonstrating the validity of Arrhenius equation (Eq. 8):

8 $$\sigma ={\sigma }_{dc}\text{exp}(-{E}_{g} /kT)$$

(*E*_g_ represents the conduction activation energy and k is Boltzmann constant).

The *E*_g_ values were calculated from the slope of the straight lines in Fig. 7 and are given in Table 3.

**Table.3:** Variation of ε’ and ε’’ at RT/100 Hz on Ag_2_O/ Li_2_O replacement mol%.

Ag_2_O/ Li_2_O replacement mol%	ɛ'	ɛ''	Frequency exponent s	Activation energy E_g_ (ev)
0	1527.6	678.02	0.67	0.36
0.25	1260.1	551.40	0.69	0.38
0.5	1094.7	490.47	0.74	0.41
1	1037.7	434.82	0.79	0.43
2	922.4	406.10	0.88	0.45
4	885.6	399.81	0.92	0.49

**Fig. 8:**
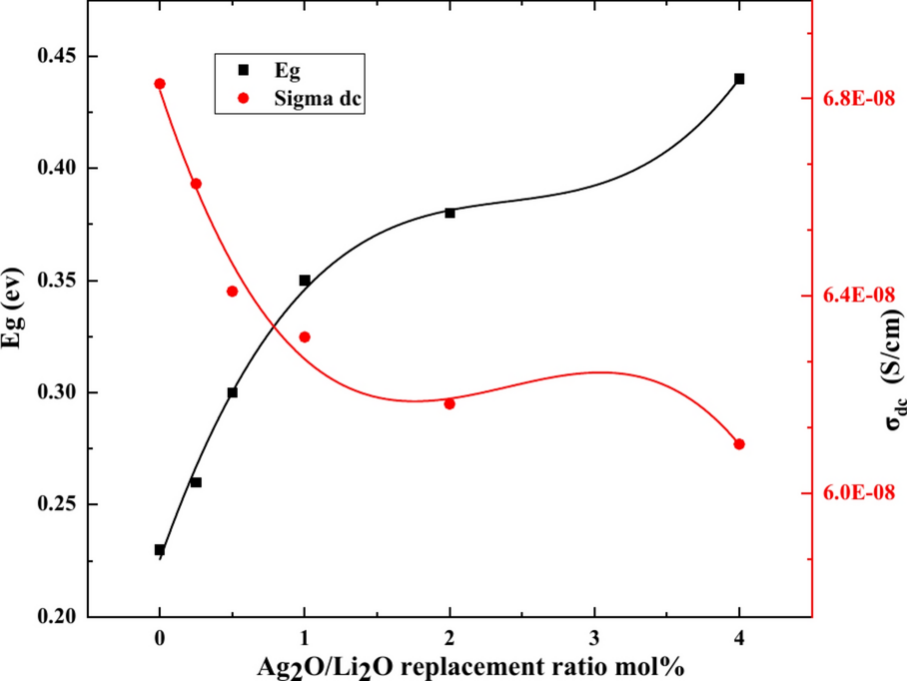
variation of activation energy E_g_ and σ_dc_ with Ag_2_O/Li_2_O replacement.

On the other hand, the *E*_g_ values were found to increase with Ag_2_O/Li_2_O replacements (Table 3). The DC conductivities at RT and the activation energy (E_g_) are plotted in Fig. 8 as a function of composition.

The enhanced values of *E*_g_ could be rationalized from the larger ionization energy required to ionize the substituted silver atoms in mixed silver and lithium ion environment. The resulting decrease in electrical conductivity could be due to the rigid structure of glass.

Additionally, the higher ionic radius along with the higher molecular weight of Ag^+^ ions were reported to restrain the transit of Li^+^ions inside the glass matrix^58,59^.

The frequency dependent conductivity σ_ac_ (ω) increases approximately linearly with angular.

frequency ω following Eq. 7.

**Fig. 9:**
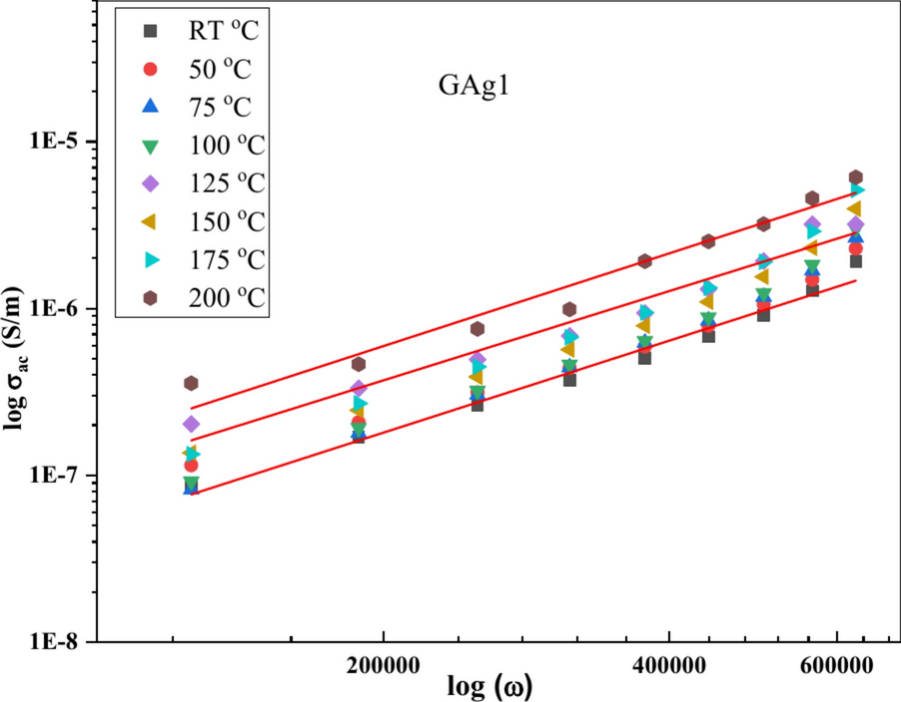
Variation of Log σ_ac_ versus Log (ω) at different temperatures for GAg1.

We can obtain the “*s*” values by fitting the slope of the linear log (*σ*_ac_) vs. log (*ω*) Fig. 9, lying in the range of 0.67–0.92 for all samples at RT (Table 3) which is typical of conductors in Jonscher regime^60^.

The s value exhibited an upward trend as the Ag_2_O content increased (Table 3). This trend suggested a reduction in the available free space for Li^+^ and Ag^+^ ion diffusion as Ag_2_O concentration rose^61^. Structural studies revealed that samples with higher Ag_2_O concentrations experienced a decrease in glass molar volume. This increased density hindered the easy movement of conductive species such as Li^+^ and Ag^+^ ions, resulting in a decrease in conductivity.

In fact, if we check the behavior of sample GAg1 (with no silver), we could notice that the ionic conductivity seems to be prevailed, and we can conclude from this observation that the contribution of Li^+^ ions in conductivity could not be excluded. Whereas in case of higher Ag_2_O, containing samples, a prevalence of ionic conductivity is also noticed which is attributed to the high diffusion of both Ag ^+^ and Li ^+^ ions. And these results were also supported by the literature reports mentioned in ref^58^.

**Fig. 10:**
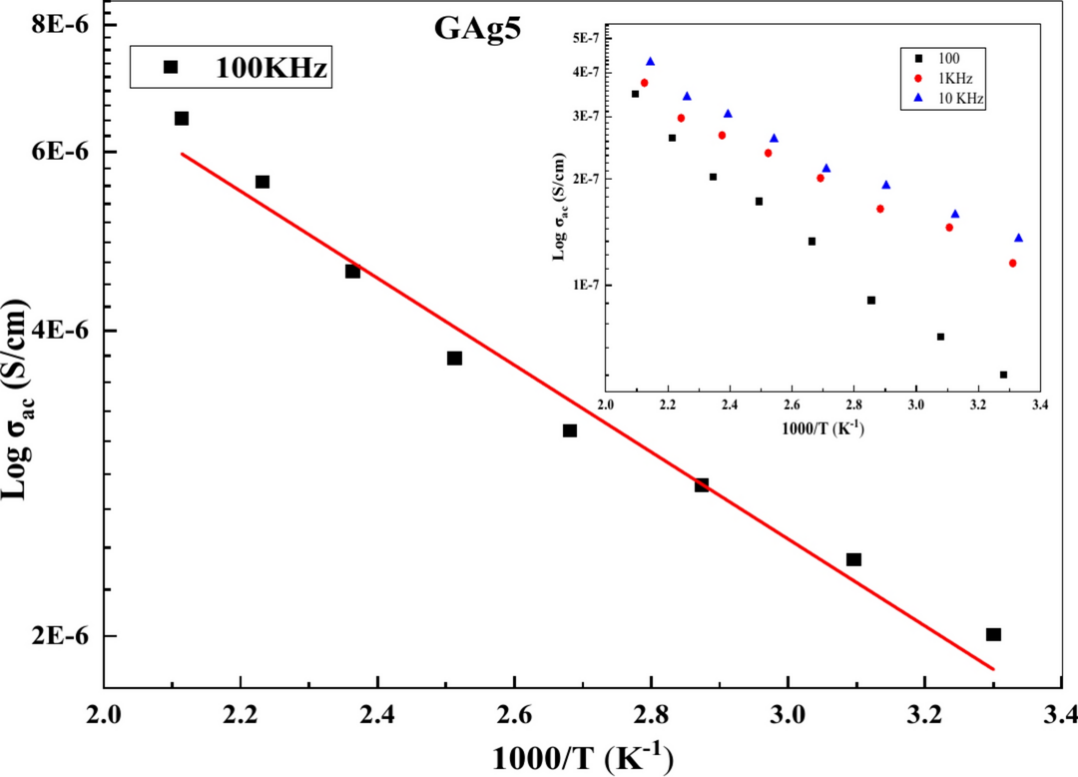
Plots of log σ_ac_ vs. 1000/T at different frequencies for GAg5 sample.

Figure 10 illustrates the temperature-dependent conductivity at various frequencies. The graph shows a linear increase in σ_ac_ with the inverse of absolute temperature, demonstrating that AC conductivity is thermally activated. Furthermore, the behavior follows an Arrhenius-type pattern.

**Fig. 11:**
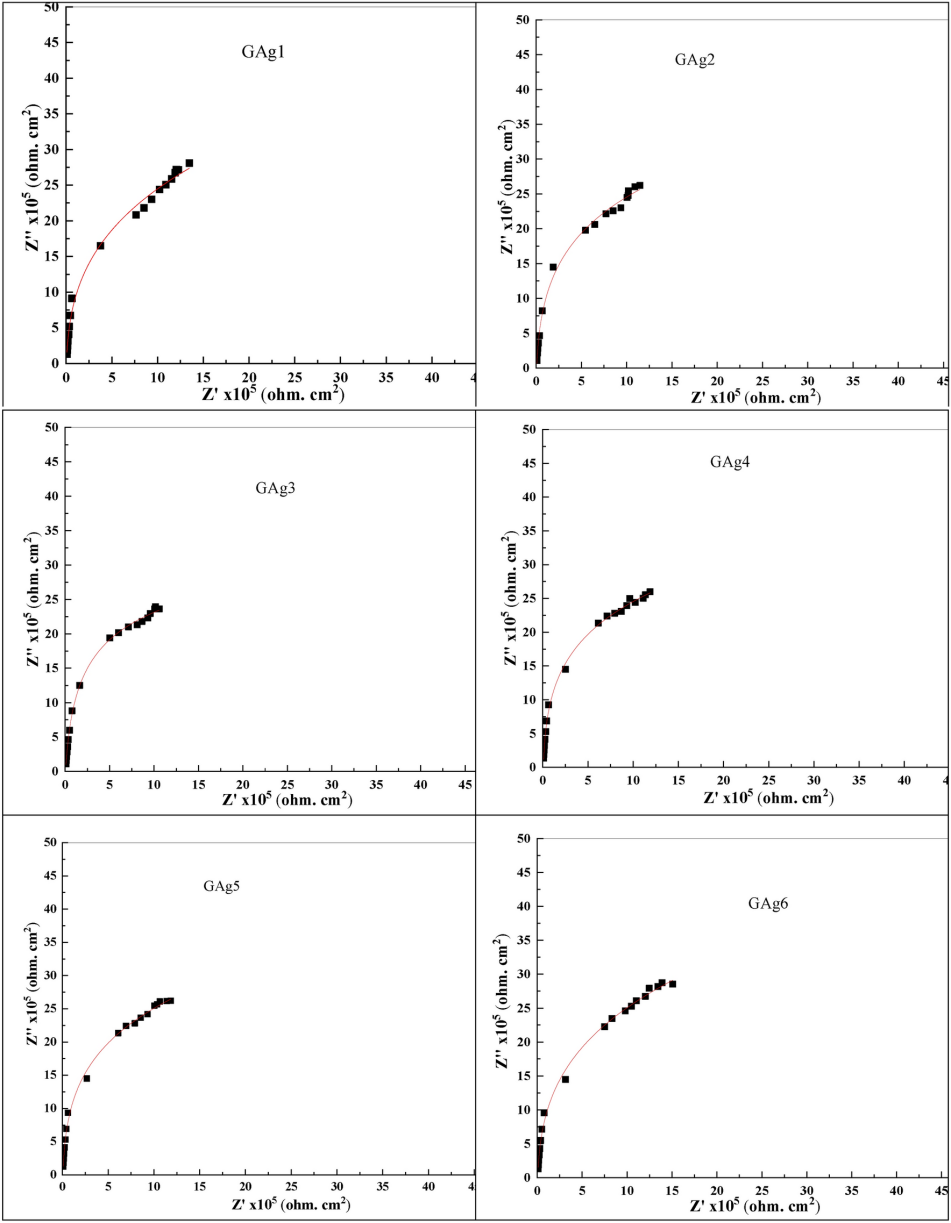
Experimental Nyquist plots for all glass samples at room temperature.

In addition, for our studied glasses, the electrical impedance (Z*) was evaluated through plotting the imaginary (Z'') versus real (Z') parts at RT as shown in Fig. 11 which exhibited such impedance plot (known as Nyquist plots) for all glass samples. For all glasses, we can observe only the beginning of the semicircle as due to the limit of the instrument.

A part of straight line was observed in the spectra of samples (GAg1-GAg6), which is an evidence of generation of interfacial impedance produced from charge carrier’s accumulation at the blocking electrode on the sample^3^. Additionally, with Ag_2_O/ Li_2_O replacements in glass samples, an increase of the semicircle diameter as well as shifting of their intersection points to higher Z’ values were observed which suggests the increasing value the bulk resistance R_b_ of the sample and consequently the conductivity decreases.

#### Frequency dependent dielectric properties

The dielectric constant (ε) of the samples was calculated from the basic Eq. 9.

9 $$C=\frac{\varepsilon {\varepsilon }_{0}S}{d}$$

where C, S, d represent capacitance, area, and thickness of the sample respectively, while ε_o_ denotes the free space permittivity.

**Fig. 12:**
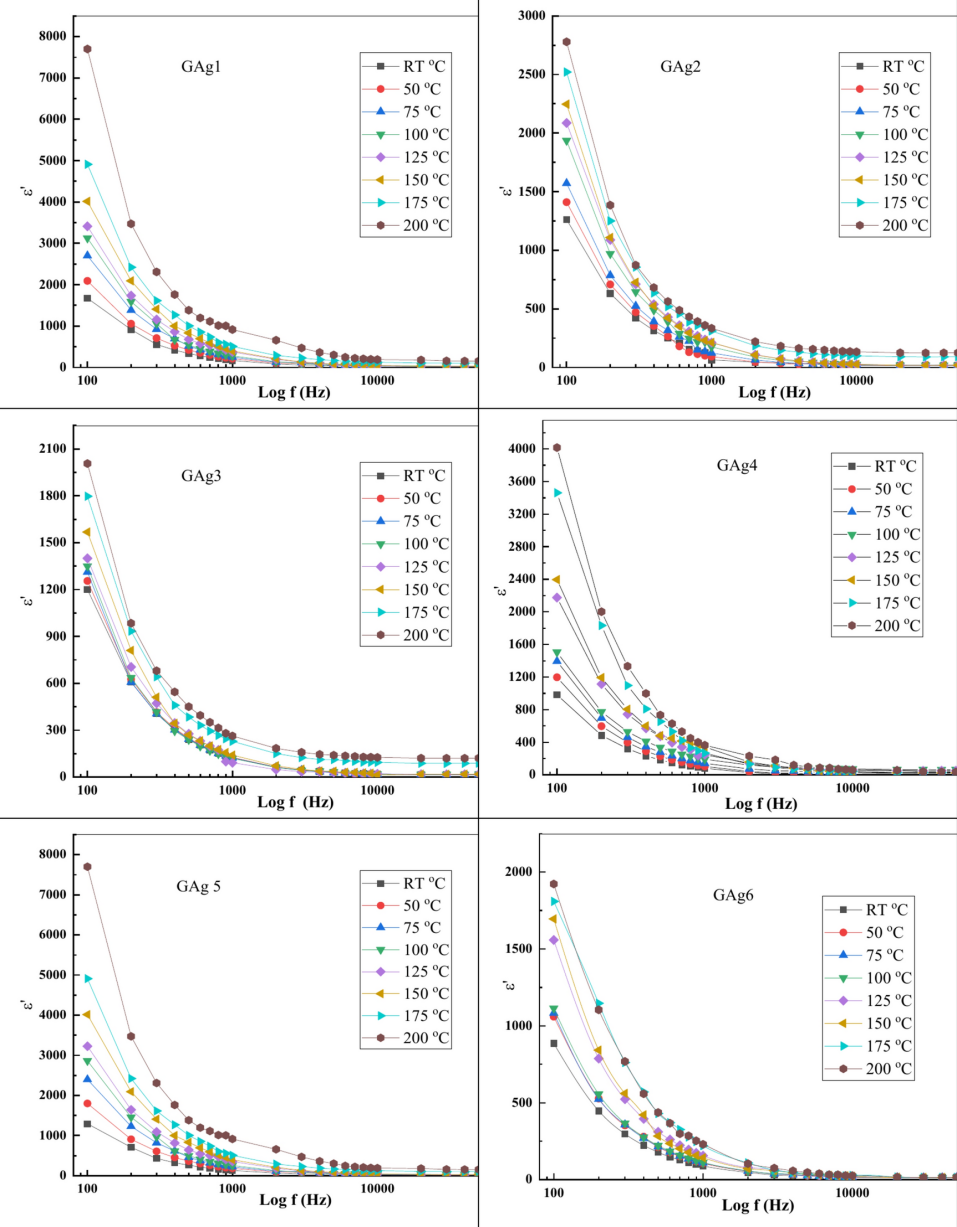
Plot of ε' as a function of (f) for the glass samples at different T.

The plotted curves of ε' as a function of (f) for glass samples (GAg1, GAg2, GAg3, GAg4, GAg5 and GAg6) in a temperature range of (RT–200 °C) is shown in Fig. 12.

We can notice that at low frequencies, there is an inverse relationship between ε' and frequency, while it increases with temperature. In fact, this behavior was observed in previous studies^62,63^. Specifically, beyond frequencies 10^3^ Hz, there is a weak dependence of ε' on frequency/temperature. Tareev explained this behavior based on the faster response of charge carriers with the external applied electric field (E_ex_) at low frequencies generating an increase of *ε*′^64^. While with expanded high frequencies the charge carriers fail to follow the rapid changes in (E_ex_) generating a decrease of *ε*′ and this decrease remains unchanged and approaches constant value with higher frequencies.

**Fig. 13:**
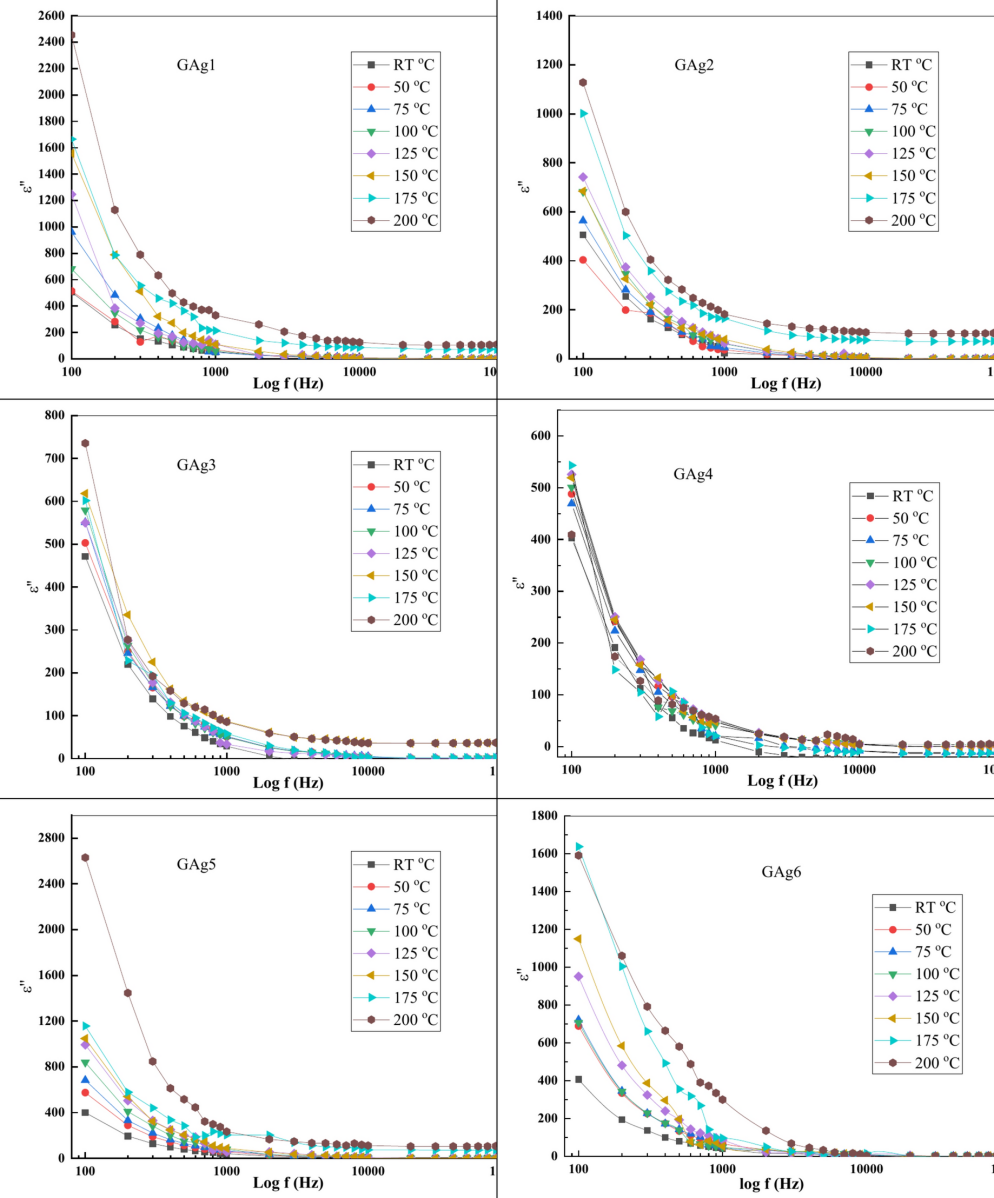
Plot of ε'' as a function of (f) at different temperatures.

Loss factor (ε") as a function of (f) for glass samples (GAg1, GAg2, GAg3, GAg4, GAg5 and GAg6) in the temperature range of (RT–200 °C) was calculated using Eq. (10) is shown in Fig. 13.

10 $${\varepsilon }^{{\prime}{\prime}}={\varepsilon }^{\prime}\text{tan}\delta$$

Here tan δ (δ = 90- φ) is the loss tangent and φ is the phase angle obtained directly from measurements.

Loss factor (ε") values are found to have the same trend as ε' which increases with increasing T and has a rapid decrease at low f, while being almost constant at high f. In Fig. 13, the factor loss behavior as a function of f could be rationalized from the migration of ions within the material at low f. The values of ε" at moderate f are due to the charity of ion jump and conduction loss of ion migration, in addition to the ion polarization loss. At high f, ion vibrations may be the sole source of the dielectric loss, hence *ε*″ is f-independent^65^.

#### Temperature and compositional dependence

**Fig. 14:**
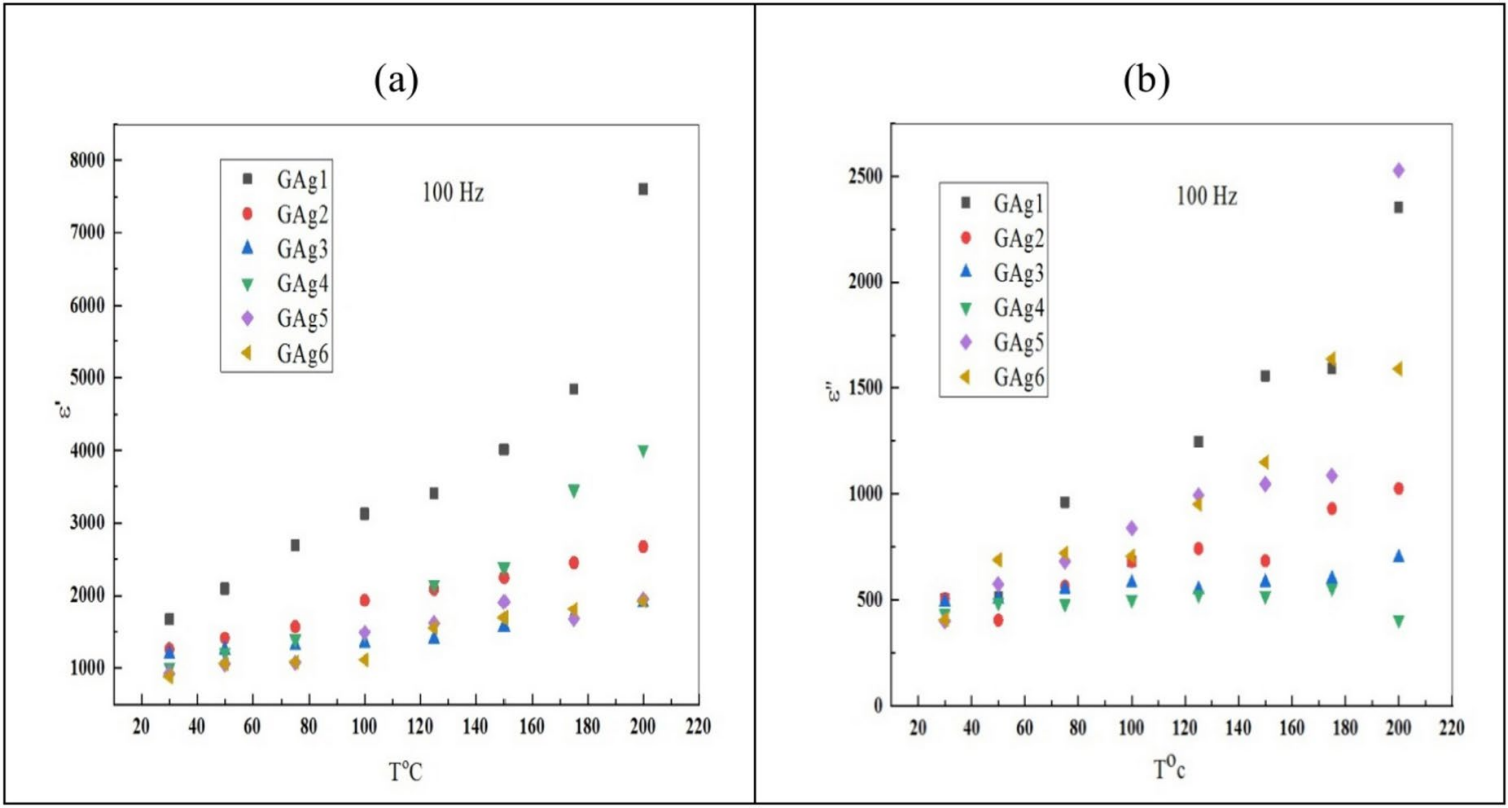
Temperature dependence of ε' (a) and ε'' (b) for all glass samples at 100 Hz.

Figure 14a,b shows the T dependence of ε' and ε'' for all the studied glass samples at 100 Hz. The observed increase in ε' with the increase of T could be attributed to the charge carriers’ contribution to the polarization^66^. At low T, the dipoles become unable to rotate fast enough and this weakens the polarization; consequently, the dipoles oscillate behind the field. On the other hand, the increase of T generates a sufficient thermal excitation energy gained by the bound charge carriers, which improves the polarization resulting in the increase of the dielectric constant. The ε'becomes larger at lower f and higher T which is the usual behavior for oxide glasses. On the other hand, ε'' display significant frequency dependence at higher T. The root of ε'' was proposed to include dipolar and conduction losses, in addition to the vibrational losses^67^. However, increasing the T of the samples leads to an increase of the electrical conduction losses accompanied by an increase of ε''.

We also studied the effect of compositional variation of the studied samples on the dielectric parameters (ε' and ε'') at (RT/100 Hz) as presented in Table (3).

**Fig. 15:**
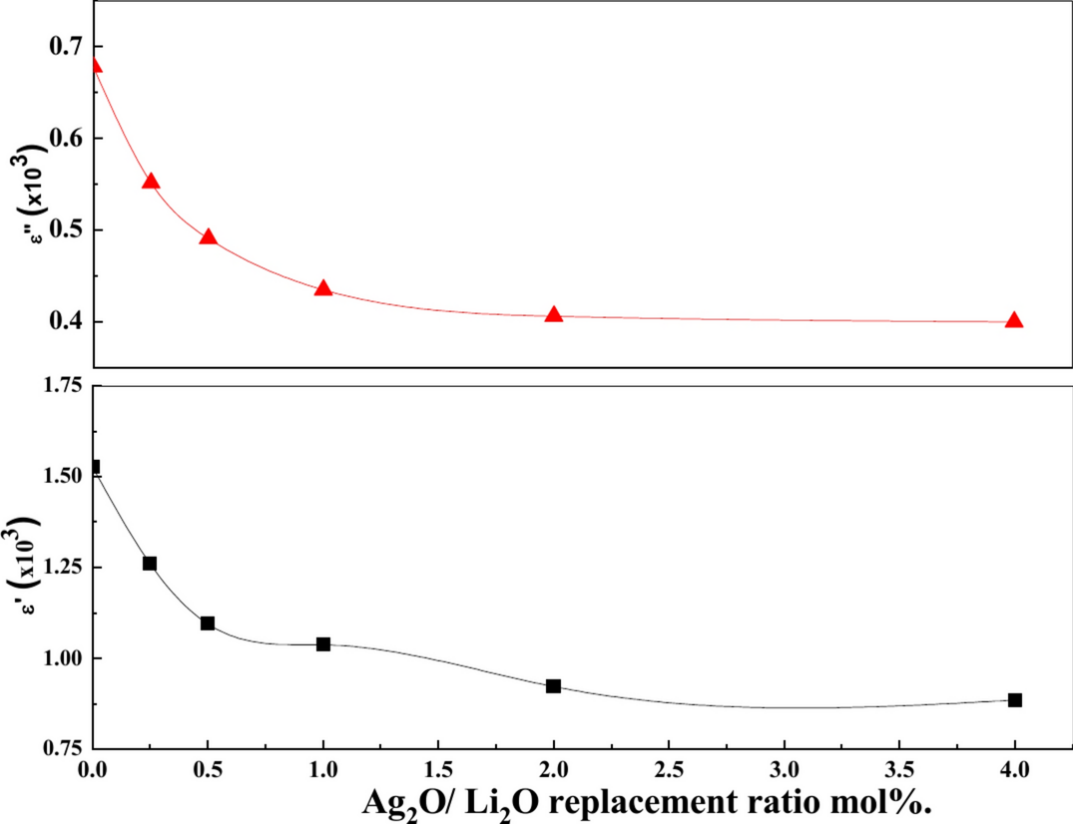
Composition dependence of dielectric constant ε' and ε'' for all glass samples at RT/100 Hz.

The results revealed that the increasing of Ag_2_O/Li_2_O replacement caused a decrease in values of dielectric parameters (ε' and ε'') Fig. 15. This decrease could be interpreted on the basis of the change occurred in the internal network of the glass structure. The infrared spectral and molar volume measurements of these samples showed that Ag_2_O addition to the studied samples increase the strength of the glass structure and the bridging oxygen instead of non-bridging oxygens (NBO). This causes retardation for the charge carriers migration responsible for the space charge polarization occurred at the electrodes, and consequently decrease the dielectric parameters value^67^.

## Conclusions

A series of glass specimens based on the lithium silver magnesium bismuth silicate system with the general formula (20-Y) Li_2_O-YAg_2_O–20MgO–10Bi_2_O_3_−50SiO_2_ (Y = 0.5, 1.0, 2.0 and 4.0 mol %) was prepared. The replacement of Li_2_O by Ag_2_O meaningfully affected the physical and electrical properties of the prepared glass materials. The Ag_2_O/Li_2_O replacement led to an increase of density, oxygen packing density, and field strength with decreasing molar volume values for the prepared glasses. The results of the physical parameters indicate that the increase in compactness of the glass structure with the replacement of Li_2_O by Ag_2_O caused a decrease in the values of dielectric parameters, while the activation energy for a.c. conduction was found to increase.

## Data Availability

The authors declare that the data supporting the findings of this study are available within the paper. Should any raw data files be needed in another format they are available from the corresponding author upon reasonable request.
